# Lip and Oral Cavity Cancer Burden and Related Risk Factors in China: Estimates and Forecasts from 1990 to 2049

**DOI:** 10.3390/healthcare10091611

**Published:** 2022-08-24

**Authors:** Jingya Zhang, Yongbo Lu, Haoran Li, Ning Zhang, Rongxin He, Ruhao Zhang, Ying Mao, Bin Zhu

**Affiliations:** 1 School of Public Policy and Administration, Xi’an Jiaotong University, Xi’an 710049, China; 2 Vanke School of Public Health, Tsinghua University, Haidian District, Beijing 100084, China; 3 School of Public Health and Emergency Management, Southern University of Science and Technology, Shenzhen 518055, China

**Keywords:** lip and oral cavity cancer, China, joinpoint regression, GBD study, risk factors, age-period-cohort model

## Abstract

**Simple Summary:**

China’s lip and oral cavity cancer burden is rising. The elderly have a relatively heavy disease burden, mainly due to poor oral health awareness, the side effects of other diseases and delayed treatment. Moreover, the incidence of the elderly over 50 years old is predicted to increase further from 2020 to 2049 in China. Males have a heavier disease burden, mainly due to their smoking, drinking and work exposure. Early screening and health intervention policies incorporating key populations and risk factors may deserve the consideration of policy makers to reduce the disease burden.

**Abstract:**

Lip and oral cavity cancer is a common malignancy faced by many developing countries, and the disease burden is high in China. This study explored this cancer burden and its risk factors using data from China in the GBD 2019, along with predicting the incidence trends in 2020–2049. Data on age-standardized rates (ASR), incidence, death and disability-adjusted life years (DALY), by sex, age and risk factors were collected from the Institute for Health Metrics and Evaluation (IHME). Joinpoint regression and Age–Period–Cohort (APC) models were selected to analyze the epidemic trend of this cancer in China, and descriptive analysis was used for the time trend and age distribution of risk factors. The Bayesian APC model was selected to foresee the incidence trend in 2020–2049. This cancer burden was found to be in an upward trend in China in 1990–2019. The upward trend was more pronounced among men than among women. These cancer deaths and DALYs are overwhelmingly attributable to smoking and drinking. On APC analysis, the younger generation in China demonstrated a lower cancer risk. In 2049, the incidence of this cancer is projected to be 3.99/100,000, 6.07/100,000, 7.37/100,000, 10.49/100,000, 14.82/100,000, 19.19/100,000, 20.71/100,000, 23.64/100,000, 16.42/100,000 and 9.91/100,000 among those aged 50–54, 55–59, 60–64, 65–69, 70–74, 75–79, 80–84, 85–89, 85–89 and over 95 years, respectively. Disease control policies and early screening should focus on men and the elderly and target different risk factors.

## 1. Introduction

Lip and oral cavity cancer is a serious problem in the world, and is closely related to national health [1]. Human head and neck cancers can originate in multiple body sites, including the salivary glands, sinuses, throat, mouth, larynx, or nose. Lip and oral cavity cancer is a subgroup of these cancers [2] and can occur in any part of the mouth [3]. The predominant form of this cancer in most cases is squamous cell carcinoma [4].

Due to its high mortality rates and side effects, it is considered a critical warning to human health [5]. As the prognosis of this cancer is associated with the local economy and medical facilities, it has a poor prognosis in developing countries and often brings a severe disease burden to patients and society [6]. It is the fourth most widespread cancer in a large number of low- and middle-income countries and has the lowest five-year survival rate [7] and the sixth highest mortality rate [8]. Despite current advances in the therapy of this cancer, population-based survival studies have not shown significant developments in survival over the past few decades [9]. Early diagnosis and prevention remain pivotal for improving the survival rates. The World Health Organization has considered necessary actions to control this disease as a fitness priority [10].

There are many risk factors for this cancer [11], including lifestyle factors, such as the excessive use of tobacco [12,13], alcohol consumption [14], paan (betel quid chewing) [15], exposure to sunlight [16] and diet [17,18,19]; hygiene factors, such as oral and dental health [12] and the use of mouthwash [14]; personal physical factors, such as Body Mass Index (BMI) [20], race [13,21] and genetic factors [13,22]; disease factors, such as oral mucosa disease [14], human papilloma virus infection [13,23], immunosuppression and immunodeficiency [21]; and socioeconomic factors, such as occupational exposure [13], place of residence [24] and social inequality [13,14,21]. At the same time, marijuana use [25] and unsafe sex [24] are also considered risk factors. Emerging research [26,27,28,29] suggests that some risk factors associated with this cancer, including smoking, obesity rates and drinking, have risen markedly during the COVID-19 pandemic. Outcomes of this disease may be affected by the COVID-19 pandemic for decades [30].

The incidence and mortality of this cancer demonstrate a wide geographical variation worldwide, especially among developing and developed countries [31]. In particular, as a country severely affected by it, China had the third highest incidence of this cancer worldwide and the second highest number of deaths in 2012 [11]. To make matters worse, about 30,117 cases of this cancer and 14,285 deaths were reported by the International Agency for Research on Cancer in 2020. China remains the country with the second highest death toll in the world; however, its rank with respect to the number of cases has risen from third to second. However, to date, few studies have concentrated on this disease burden in China.

To fill this void, we dedicated our research to analysis of the disability-adjusted life years (DALYs), deaths, incidence and risk factors of lip and oral cavity cancer in China during 1990–2019. We also managed to forecast the trend of its incidence in China in the next 30 years. The findings might inform pertinent health strategy and be conducive to the effective allocation of medical resources to prevent and control this disease.

## 2. Methods

### 2.1. Data Source

The Crude Incidence Rate (CIR), Crude Death Rate (CDR), Crude DALY rate, Age Standardized Incidence Rate (ASIR), Age Standardized Death Rate (ASDR) and age-standardized DALY rate related to this cancer were searched by sex and age in the global burden of disease 2019 (GBD 2019) database (https://vizhub.healthdata.org/gbd-results/) (accessed on 18 May 2022) [32,33,34,35] from 1990 to 2019. Data on the three main risk factors (smoking, alcohol use and chewing tobacco) for ASDR and age-standardized DALYs were collected by sex and age group [36]. The GBD 2019 database, developed by the Institute for Health Metrics and Evaluation (IHME) at the University of Washington, provides canonical and comparable measurements of vital health issues on a global scale so that sanitation systems can be ameliorated and health gaps can be reduced [37,38].

Matrix data of the population were collected from the United Nations Population Division’s World Population Prospects (2019 Revision) (https://population.un.org/wpp/Download/Standard/CSV/) (accessed on 19 May 2022) for morbidity projections [39]. This report collects and predicts the total population of different countries and regions in the world from 1950 to 2100.

### 2.2. Statistical Analysis

**Figure 1:**
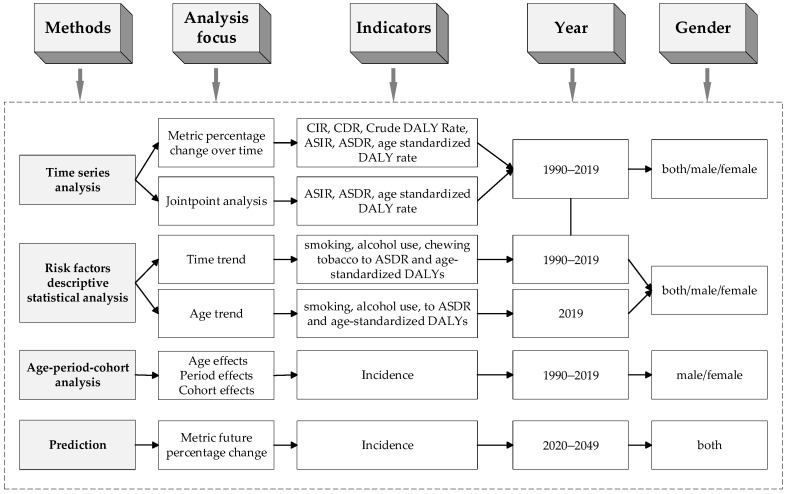
Analysis roadmap of this study.

Figure 1 shows the analysis roadmap of this study. The joinpoint regression methods were used to evaluate the time trends and their significance [40]. This allows for characterization of the data trend throughout the observation period and calculates the specific timepoint when the trend changed [41]. To examine the changes in the Age-Standardized Rate (ASR), the annual percent change (APC) was reported by using a joinpoint regression model of the natural log-transformed rates with the selected joinpoints. To judge the orientation and rangeability of the overall trends in this cancer, the average APC (AAPC) values from 1990 to 2019 were also assessed. In this study, the natural logarithm of ASR was selected as the response variable, and the notification year was used as the independent variable (See Appendix A).

We performed a descriptive analysis of both the temporal and age trends of risk factors for this cancer in China. GBD 2019 reported the contribution of smoking [12,13], alcohol use [14] and chewing tobacco [15] to ASDR and age-standardized DALYs of this cancer. Therefore, we analyzed the time trend of the contribution of these three risk factors from 1990 to 2019. As for the Chinese data, smoking and alcohol use were closely linked to ASDR and age-standardized DALYs of this disease compared to chewing tobacco. Therefore, we focused on the age difference between these two risk factors in 2019.

According to GBD 2019 data, the incidence of lip and oral cavity cancer in people over 50 years old is higher than the average of all age groups (3.1790/100,000) in China. Therefore, we conducted age-period-cohort (APC) model analyses for men and women over 50 years separately and combed the original data based on model requirements. The first step was to divide people aged over 50 years into 10 groups (50–54, 55–59, 60–64, 65–69, 70–74, 75–79, 80–84, 85–89, 90–94 and 95+ years) that were five years of age apart. Secondly, during the whole observation period from 1990 to 2019, the same 5-year interval was divided into six groups (1990–1994, 1995–1999, 2000–2004, 2005–2009, 2010–2014 and 2015–2019). Finally, 15 birth cohorts (1892–1896, 1897–1901, 1902–1906……1952–1956, 1957–1961 and 1962–1966) were obtained by subtracting age from the period. (Details can be found in Supplementary Materials Table S1)

We calculated the age, period and cohort effects separately by using natural logarithm of disease incidence as the dependent variable and selecting median of these datasets as the independent variable. As age, period and cohort have a completely linear relationship, there is a problem that the model cannot be recognized. In order to overcome this multicollinearity problem, the intrinsic Estimator (IE algorithm) was used in this study. This statistical approach is also widely used in published papers on global epidemics regarding the incidence rates of many diseases [42,43,44] (See Appendix B).

As demographics change and treatments and diagnoses evolve, there is a growing interest in age-stratified cancer incidence rates [45]. Bayesian APC models are well-fit for analyzing predictions of age-stratified cancer incidence. Therefore, we predicted the incidence with regard to the changing character of the population over 50 years old from 2020 to 2049 using the Bayesian APC model. The population was grouped into 10 subsets of 50–54, 55–59, 60–64, 65–69, 70–74, 75–79, 80–84, 85–89, 90–94 and 95+ years. Based on the 30-year (1990–2019) time series data of the incidence of this disease in China (Details can be found in Supplementary Materials Table S2) and the 30-year (1990–2019) time series data of the population in China (Details can be found in Supplementary Materials Table S3), the incidence in the 10 age groups was predicted (See Appendix C).

### 2.3. Software

The Joinpoint Regression Program (version 4.9.0.0) was employed to analyze the trend of ASIR, ASDR and age-standardized DALY rate changes of this cancer from 1990 to 2019. For APC model analyses and graphs, APC fit in Stata (version 13.0) was used. The Bayesian APC was modeled using BAMP package in R (version 4.1.12) to predict the incidence in the next 30 years (2020–2049). All figures were drawn by using OriginPro (version 2020b).

## 3. Results

### 3.1. Lip and Oral Cavity Cancer Burden in China

**Figure 2:**
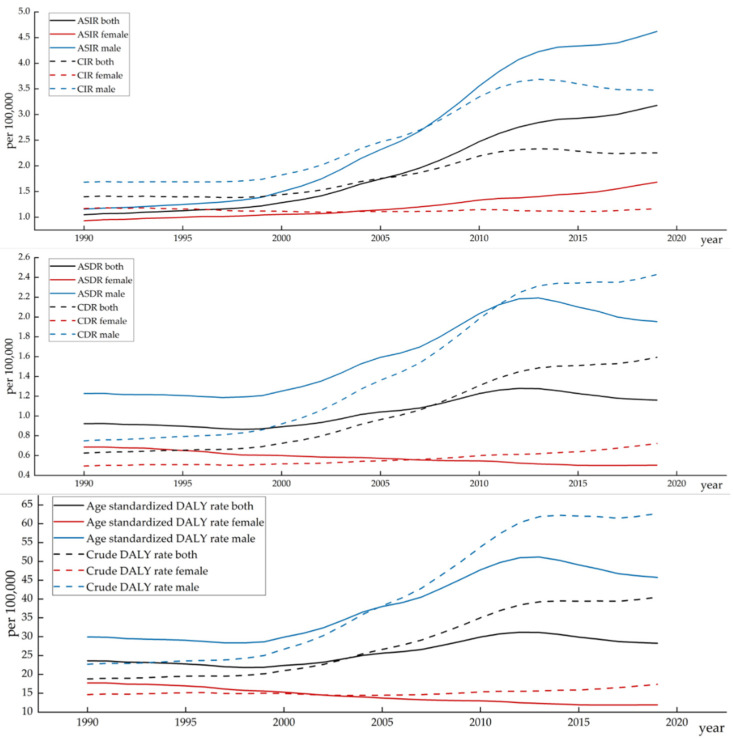
Trends of lip and oral cavity cancer burden in China from 1990 to 2019.

Figure 2 shows the tendency of deaths, incidence and DALYs of lip and oral cavity cancer by gender in China from 1990 to 2019. Overall, an upward trend was noted for incidence, deaths and DALYs of this cancer between 1990 and 2019. Interestingly, the upward trend slowed down after age standardization. In detail, the CIR, CDR and crude DALY rates of this cancer have risen dramatically by 203.70%, 154.52% and 115.22% (46.88%, 42.68% and 33.37% worldwide), respectively, over 1990–2019; Apart from that, the ASIR, ASDR and age-standardized DALY rates increased by 61.03%, 25.79% and 19.63% (5.48%, 0.03% and −1.43% worldwide), respectively, from 1990 to 2019.

From the perspective of sex, the burden of the disease was more serious among men. Likewise, the growth rate of the disease was higher among men compared to women. Among men, as with the trend of the total population, the CIR, CDR and crude DALY rates of this cancer increased by 299.47%, 224.31% and 176.24%, respectively, throughout the study period, while the ASIR, ASDR and age-standardized DALY rates went up by 106.66%, 59.34% and 52.7%, respectively, from 1990 to 2019. Among women, on the contrary, the CIR, CDR and crude DALY rates of this cancer increased by 80.89%, 46.11% and 18.38% in 1990–2019, respectively. The ASIR, ASDR and age-standardized DALY rates witnessed a downward tendency, decreasing by 0.75%, 26.64% and 32.73%, respectively, from 1990 to 2019. (Details can be found in Supplementary Materials Table S4)

### 3.2. Joinpoint Regression Analysis of the Disease Burden of Lip and Oral Cavity Cancer in China

Table 1 shows the results of the Joinpoint Regression Analysis for this disease in 1990–2019. The ASIR was growing steadily (AAPC = 1.6) and at first climbed dramatically from 2001 to 2012, followed by a slightly downward change. Among men, similar to the result of the whole population, the ASIR increased gradually throughout the entire research period (AAPC = 2.5), consisting of an upward trend at the start from 1999 to 2012 and a slightly downward trend afterwards from 2012 to 2019. Among women, the ASIR demonstrated a fluctuating pattern (AAPC was approximately equal to 0). A decreasing trend was found in the study period from 1993 to 2001, as well as from 2010 to 2016, while an increasing trend was detected from 2016 to 2019.

Regarding ASDR, it showed an incline in general (AAPC = 0.7). In 1990, it started to rise until 1999, followed by a downward trend from 1999 to 2012 and then rose again until 2019. Among men, likewise, the ASDR gradually increased overall (AAPC = 1.6); it went down at first until 1999 when it began to increase dramatically until 2012, followed by a slightly downward trend. Among women, the ASDR notably showed a decreasing trend from 1993 to 2015 (AAPC = −1.1).

Lastly, a slight growth in the age-standardized DALY rate appeared to be the result of a rising trend (AAPC = 0.6) from 1999 to 2012; however, the rising trend began to lose momentum and a downward trend kicked in from 1990 to 1999 and 2012 to 2019. Similarly, the overall progress of the indicator for the male population demonstrated an increase (AAPC = 1.5). The changing pattern among men was the same as that in the overall population. Among women, the age-standardized DALY rate demonstrated a declining trend (AAPC = −1.4).

### 3.3. Difference in Attributable Risk Factors

**Figure 3:**
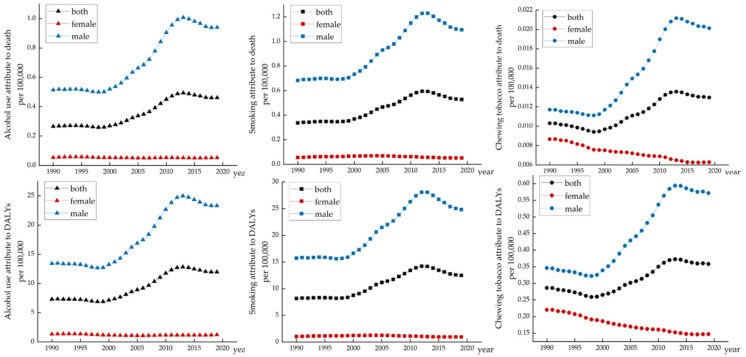
The variation trends of ASDR and age-standardized DALY rate of three risk factors in different genders over 30 years.

Throughout the research period, the effects of the factors increased at first and then began to decrease steadily after hitting the summit, peaking around 2014. Among the three risk factors, smoking and alcohol use were closely linked to ASDR and age-standardized DALYs of this cancer compared to chewing tobacco. The changing character among men was akin to that in the entire population. Among women, the effects of smoking and alcohol use showed a decline in a fluctuating manner. In addition, the influence of smoking on this cancer burden in the Chinese male population was greater than that of alcohol use. Among women, the impact of smoking and alcohol use were not much different (Figure 3). (Details can be found in Supplementary Materials Table S5)

**Figure 4:**
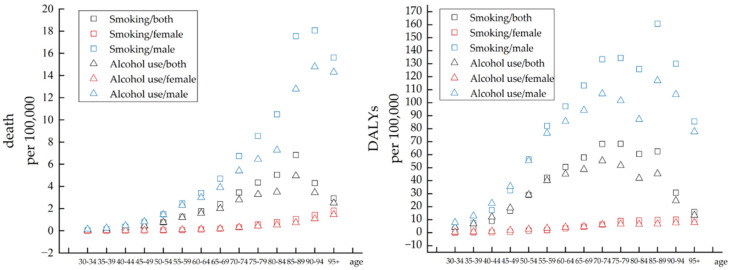
The variation trend of ASDR and age-standardized DALY rate of two risks in different genders and age groups in 2019.

From the perspective of age, the age-distribution characteristics of smoking and drinking were basically the same among men and both showed an increasing trend first, followed by a decrease with age. In the group aged between 70 to 94 years, the contribution of smoking was significantly higher than that of alcohol use. Before the age of 70 years, the difference was not apparent. Notably, the contribution of smoking and alcohol use to ASDR peaked at the age of 90–94 years, while the impact on the age-standardized DALY rate summited at the age of 85–89 years. Among women, the effects of smoking and alcohol use showed a positive association with age with no significant difference in their contributions and climaxed at the age of 95 years plus (Figure 4). (Details can be found in Supplementary Materials Table S6)

### 3.4. APC Model Analysis of Lip and Oral Cavity Cancer Incidence in China

**Figure 5:**
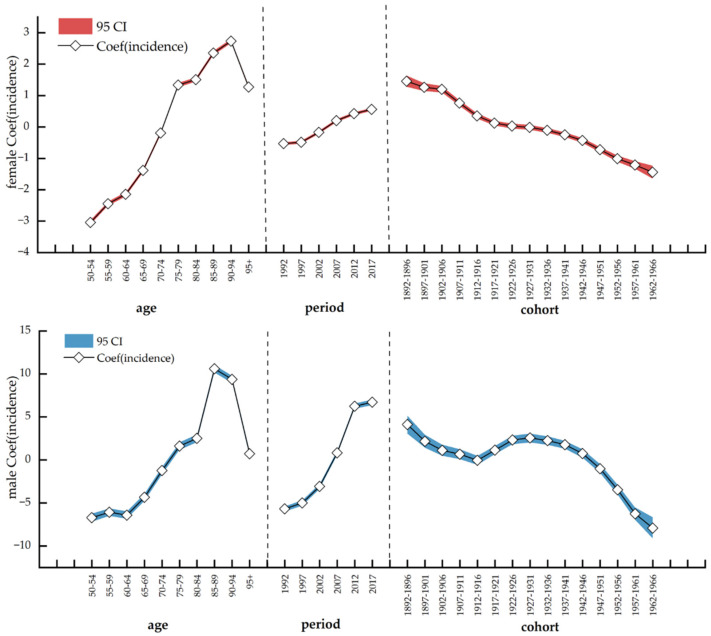
APC model analysis of lip and oral cavity cancer incidence among females and males in China. Note: APC model, Age–Period–Cohort model; Coef: coefficient; and CI: confidence interval.

As shown in Table 2 and Figure 5, the age effect on lip and oral cavity cancer incidence showed an increase in China. Likewise, the period effect showed a rising trend along with time, while the cohort effect went down throughout the timeline. The age effect coefficient for both sexes grew from the start and then gradually declined after reaching the apex. For people aged under 75 years, the age effect coefficient for women was larger than that for men, while for individuals over 79 years of age, the age effect coefficient for men was greater than that for women. However, when the indicator hit 85 years, the effect coefficient for men decreased significantly, while the downward trend among women was less significant.

The period effect coefficients for both sexes showed an upward trend. Besides, the growth rate in men was greater. Before 2005, the period effect coefficient for women was larger than that for men; conversely, after 2005, the period effect coefficient for men was more considerable than that for women. The cohort effect coefficients for both sexes showed a decreasing trend. In addition, the decline rate in men was more pronounced. Before 1950, the cohort effect coefficient for men was greater than that for women; however, after 1950, the cohort effect coefficient for women was larger than that for men (Figure 5).

### 3.5. Prediction Based on the Bayesian Age Cohort Model

**Figure 6:**
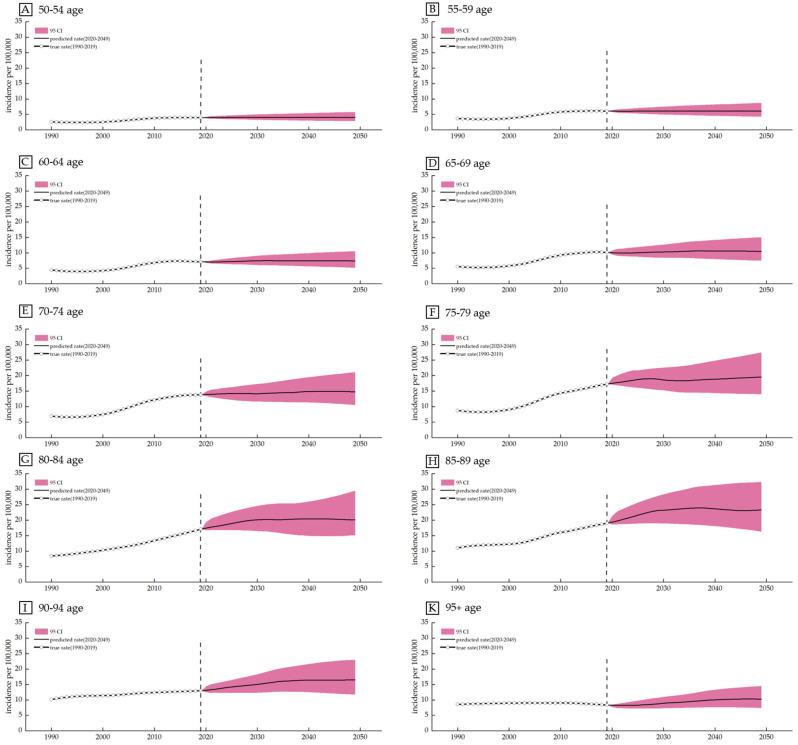
Prediction of lip and oral cavity cancer incidence among females and males in China from 2020 to 2049 based on the Bayesian APC model.

The results showed a slightly upward trend in every age group. The incidence in the 50–54 years age group is projected to rise from 3.81/100,000 in 2019 to 3.99/100,000 in 2049, with a growth rate of 4.71%. For the 55–59 years age group, the incidence will slightly increase from 5.85 per 100,000 in 2019 to 6.07 per 100,000 in 2049, with a growth rate of 3.79%. The rates of increase are 4.84% (60–64 years), 8.47% (65–69 years), 11.37% (70–74 years), 21.84% (75–79 years), 39.84% (80–84 years), 37.44% (85–89 years), 27.34% (90–94 years) and 12.02% (95+ years) for the individual age groups (Figure 6). (Details can be found in Supplementary Materials Table S7).

It is noteworthy that the smallest growth rate of 3.79% was detected in the 55–59 years age group, while the largest rate of increase was at the age of 80–84 years. Furthermore, apart from the group aged above 95 years, the higher age groups tended to show more obvious trends.

## 4. Discussion

Based on the GBD 2019 data, we conducted trend analysis and prediction of the lip and oral cavity cancer burden and its related risk factors in China from 1990 to 2049. The elderly were found to be at high risk, possibly due to lower personal hygiene awareness, side effects of other systemic diseases and delays in seeking medical attention. Younger birth cohorts had a lower risk, possibly related to increased health awareness and better hygiene. Likewise, according to the forecast results by age group, the incidence will continue to show an upward trend in 2020–2049, with the upward trend in the elderly group becoming more obvious. In addition, men had a higher risk, possibly because they smoke and drink more, and men’s occupations make them more likely to be exposed to dangerous chemical factors and drinking subcultures.

This study also found that early screening may be responsible for the rising incidence of this disease in China during the study period. In the long term, early screening and health intervention policies can reduce the disease burden. From the perspective of age, the unreasonable population structure in China may be a significant reason for the increased burden of this cancer compared to other countries. The proportion of Chinese residents over 65 years is increasing, and the birth rate is declining with the overall population showing an aging trend [46].

According to the GBD data in 2019, the disease burden of this cancer in China increased marginally, and both the morbidity and mortality showed an increasing trend. After adjustment of age standardization, the growth rate of this cancer burden in Chinese men slowed down, while the growth rate of this cancer burden in Chinese women even showed a downward trend. Therefore, population aging is a potential cause of the rising burden of this cancer in China.

Global analysis [47,48] showed an increase in young patients with lip and oral cavity cancer; however, the characteristics of China differ from the epidemic trend of this cancer worldwide. This cancer burden among the elderly is heavier in China. For example, the disease burden peaks at the age of 85–94 years for men and at the age of over 95 years for women. Due to weak awareness of oral health care among the elderly, they usually fail to implement timely and effective personal oral care in daily life. At the same time, the common systemic diseases in the elderly make their oral hygiene problems more complicated [49].

Patients with delayed diagnosis had significantly greater rates of advanced this disease at diagnosis than those without delayed diagnosis [50,51]. In China, many elderly people are passive in the early stage of this cancer. For reasons such as not wanting to trouble their families, they tend to delay medical treatment [52], which also leads to a heavier disease burden in older groups. Furthermore, it is expected that the age structure of China’s population will change during 2023–2050. At that time, accelerated aging of the population will be seen. The proportion of the elderly population aged 80 and above will increase yearly [53]. It has been suggested that, as the aging of the population becomes increasingly serious, the disease burden of this cancer will face significant challenges in the future.

The cohort effect reflects that the same social change factors (such as early life conditions, social factors and social experiences) may have similar effects on people born in the same era [54]. APC analysis indicated that the younger birth cohorts are at a lower risk of this cancer. There is rising awareness of the significance of dental wellness in the younger birth cohorts [55]. With the progressive improvement in the sanitation conditions, the younger generation also enjoys better sanitation resources. According to the China Statistical Yearbook data, the proportion of dentists in China increased from 3.2% in 2002 to 5.7% in 2020. The combined effect of increased awareness of oral health among individuals and an increase in the number of dentists has resulted in a reduced risk of the disease in the younger cohort.

The prediction results showed that the incidence of this disease in China would rise by varying degrees over the next 30 years. The incidence in the elderly aged 75–94 years will increase rapidly compared to other age groups. Additionally, the incidence at the age of 50–74 years will exhibit a slightly upward trend. Prevention is imperative for decreasing the burden of this cancer [56,57,58,59]. As China has entered an aging society [53], tertiary prevention must be integrated into the early health intervention of this cancer.

From the perspective of sex differences, the disease burden in men was found to be significantly higher than that in women, consistent with the existing research results [60,61,62,63]. Drinking and smoking are two key risk factors that cannot be ignored [64,65,66,67]. The contribution of smoking and alcohol use to this cancer in men is much greater. What’s more, the gap between men and women shows an increasing trend. Data on smoking and alcohol use in China also corroborate this result.

According to the “China Smoking Harmful Health Report 2020” issued by the National Health Commission, in 2018, there were 308 million smokers aged 15 years and above in China, including 296 million men and 11.8 million women. The “Scientific Research Report on Dietary Guidelines for Chinese Residents (2021)” showed that the alcohol drinking rate in men was 64.5%, and that in women was 23.1%, based on the monitoring results in 2015. This shows that there are more smokers and harmful drinkers among men than among women in China.

At the same time, occupational and physiological factors should be considered. Occupational exposure is a potential reason that should not be underestimated. In China, since men are mostly the backbone of the family and society, they spend more time in the workplace. Occupational exposure to chlorinated solvents, oxygenated solvents, welding and other environments can extend the risk of this cancer [68]. On the other hand, there has always been a drinking subculture in China, which makes men drink more in the workplace [69]. From the perspective of physiological factors, the female body can secrete estrogen to induce endogenous protection, making women more adaptable to a high-risk environment [70].

Lip and oral cavity cancer is often preceded by a clinically visible precancerous stage, which provides an opportunity for early screening and reducing mortality [71,72]. The overall ASIR of this cancer in China is in a rising state; however, the joinpoint analysis shows that it had a significant upward trend from 2001 to 2012. This rising trend in the incidence might be associated with enhanced early screening in China [64]. The incidence increased dramatically, which was related to the formulation and implementation of cancer prevention and control policies in China.

The Outline of China’s Cancer Prevention and Control Program (2004–2010) and other policy documents were released in the same period [65]. With the effective implementation of tertiary prevention, lip and oral cavity cancer is more likely to be found at early stages, which may also be the reason for the increased incidence during this period. However, early screening and prevention must be promoted, with particular attention to older and male populations, because morbidity and mortality can be reduced from a long-term perspective [71,72]. The joinpoint analysis results also confirmed this view. After 2012, the ASIR and ASDR of this cancer in China performed a downward tendency, proving the effectiveness of early screening.

There are certain limitations of our study. There was a lack of provincial-level data, due to which, we could not perform more specific regional comparative analysis of each region in China. Some studies have analyzed the trend of local cancer burden based on data from Chinese tumor registries. In Hunan Province [73], a major province for betel nut consumption, this cancer has become an important malignant tumor that threatens the physical and mental health of men, especially urban male residents. Tobacco control and stopping betel nut advertising are considered key steps for prevention and control.

In Shandong Province [74], the incidence and mortality of this cancer are roughly similar to the average rates in China; however, the disease burden may further increase in the future. In Beijing [75], smoking and drinking may be the leading causes of this cancer in men; however, there may be other major causes of this cancer in women. However, studies on this cancer burden have not been performed in most regions. In China, the local customs of various regions are different, and additional micro-regional analyses are needed to guide disease prevention and control policies.

## 5. Conclusions

The disease burden of lip and oral cavity cancer in China is experiencing an upward trend. Compared to the young, the elderly have a relatively heavy disease burden, mainly due to their poor oral health awareness, other systemic diseases and delayed treatment. Furthermore, the incidence among the elderly aged over 50 years is projected to further increase in the years 2020–2049. Men have a heavier disease burden, mainly due to higher smoking, drinking and work exposure.

Disease control policies and early screening should focus on men and the elderly and perform various health interventions based on the risk factors (such as oral health awareness, early screening, timely medical treatment, smoking, drinking and environmental protection at work) to achieve the purpose of effectively preventing lip and oral cavity cancer at a low cost.

## Figures and Tables

**Table 1:** The log-transformed joinpoint trends of lip and oral cavity cancer ASRs by sex in China.

Measure	Sex	Trend 1		Trend 2		Trend 3		Trend 4		Trend 5		Trend 6		1900–2019 AAPC (95% CI)
		Years	APC	Years	APC	Years	APC	Years	APC	Years	APC	Years	APC	
Age-Standardized incidence Rate	Both	1990–1998	−0.2	1998–2001	2.1	2001–2012	4.3 *	2012–2019	−0.7 *	NA	NA	NA	NA	1.6 * (1.3–1.9)
	Female	1990–1993	0.1	1993–2001	−0.8 *	2001–2007	0.1	2007–2010	1.0	2010–2016	−0.5 *	2016–2019	1.6 *	−0.0(−0.2–0.2)
	Male	1990–1999	0.2	1999–2012	6.1 *	2012–2019	−1.0 *	NA	NA	NA	NA	NA	NA	2.5 * (2.4–2.6)
Age-Standardized death Rate	Both	1990–1999	−0.9 *	1999–2012	3.1 *	2012–2019	−1.6 *	NA	NA	NA	NA	NA	NA	0.7 * (0.6–0.9)
	Female	1990–1993	−0.5	1993–1998	−2.1 *	1998–2011	−1.0 *	2011–2015	−1.8 *	2015–2019	0.1	NA	NA	−1.1 * (−1.2~−0.9)
	Male	1990–1999	−0.4 *	1999–2012	4.9 *	2012–2019	−1.8 *	NA	NA	NA	NA	NA	NA	1.6 * (1.5–1.7)
Age-Standardized DALY Rate	Both	1990–1999	−1.0 *	1999–2007	2.7 *	2007–2012	3.3 *	2012–2019	−1.7 *	NA	NA	NA	NA	0.6 * (0.4–0.7)
	Female	1990–1994	−0.8 *	1994–2006	−2.1 *	2006–2010	−0.9 *	2010–2015	−1.7 *	2015–2019	0.0	NA	NA	−1.4 * (−1.5~−1.2)
	Male	1990–1999	−0.6 *	1999–2012	4.7 *	2012–2019	−1.9 *	NA	NA	NA	NA	NA	NA	1.5 * (1.3–1.6)

Notes: AAPC, Average annual percent change; APC, Annual percent change; CI, confidence interval; and NA, not applicable. * Significantly different from zero, *p* value < 0.05.

**Table 2:** APC model analysis of lip and oral cavity cancer incidence among females and males in China.

Incidence	Female		Male	
	Coef. (95% CI)	*p* > z	Coef. (95% CI)	*p* > z
Age (years)				
50–54	−3.039 (−3.129, −2.949)	0.000	−6.695 (−7.221, −6.169)	0.000
55–59	−2.442 (−2.525, −2.36)	0.000	−6.068 (−6.552, −5.583)	0.000
60–64	−2.144 (−2.228, −2.06)	0.000	−6.405 (−6.898, −5.912)	0.000
65–69	−1.384 (−1.469, −1.298)	0.000	−4.337 (−4.837, −3.838)	0.000
70–74	−0.192 (−0.278, −0.107)	0.000	−1.248 (−1.749, −0.746)	0.000
75–79	1.333 (1.247, 1.419)	0.000	1.603 (1.101, 2.105)	0.000
80–84	1.511 (1.426, 1.597)	0.000	2.488 (1.987, 2.989)	0.000
85–89	2.353 (2.269, 2.438)	0.000	10.596 (10.1, 11.092)	0.000
90–94	2.736 (2.653, 2.82)	0.000	9.363 (8.875, 9.851)	0.000
95+	1.268 (1.181, 1.355)	0.000	0.704 (0.195, 1.213)	0.007
Period (year)				
1992	−0.531 (−0.592, −0.47)	0.000	−5.69 (−6.045, −5.334)	0.000
1997	−0.485 (−0.547, −0.423)	0.000	−4.993 (−5.357, −4.629)	0.000
2002	−0.172 (−0.234, −0.109)	0.000	−3.087 (−3.452, −2.721)	0.000
2007	0.201 (0.139, 0.263)	0.000	0.829 (0.466, 1.192)	0.000
2012	0.424 (0.363, 0.485)	0.000	6.245 (5.888, 6.602)	0.000
2017	0.563 (0.499, 0.628)	0.000	6.696 (6.318, 7.074)	0.000
Cohort (year)				
1892–1896	1.458 (1.267, 1.648)	0.000	4.091 (2.974, 5.208)	0.000
1897–1901	1.267 (1.127, 1.407)	0.000	2.162 (1.344, 2.98)	0.000
1902–1906	1.201 (1.082, 1.32)	0.000	1.108 (0.41, 1.805)	0.002
1907–1911	0.76 (0.652, 0.867)	0.000	0.654 (0.026, 1.283)	0.041
1912–1916	0.353 (0.254, 0.452)	0.000	−0.059 (−0.639, 0.52)	0.842
1917–1921	0.12 (0.029, 0.211)	0.010	1.143 (0.609, 1.677)	0.000
1922–1926	0.028 (−0.067, 0.122)	0.564	2.307 (1.756, 2.859)	0.000
1927–1931	−0.013 (−0.108, 0.081)	0.781	2.54 (1.986, 3.094)	0.000
1932–1936	−0.11 (−0.202, −0.017)	0.020	2.236 (1.693, 2.778)	0.000
1937–1941	−0.248 (−0.336, −0.159)	0.000	1.752 (1.235, 2.268)	0.000
1942–1946	−0.429 (−0.523, −0.335)	0.000	0.729 (0.177, 1.28)	0.010
1947–1951	−0.721 (−0.823, −0.62)	0.000	−1.014 (−1.609, −0.42)	0.001
1952–1956	−1.011 (−1.124, −0.898)	0.000	−3.47 (−4.131, −2.809)	0.000
1957–1961	−1.212 (−1.346, −1.078)	0.000	−6.266 (−7.051, −5.481)	0.000
1962–1966	−1.442 (−1.665, −1.22)	0.000	−7.913 (−9.214, −6.612)	0.000
Constance	5.795 (5.759, 5.83)	0.000	13.704 (13.497, 13.91)	0.000

Note: APC model, Age–Period–Cohort model; Coef: coefficient; and CI: confidence interval.

## Data Availability

The disease data used in this study are openly available in GBD 2019 at https://vizhub.healthdata.org/gbd-results/ (accessed on 18 May 2022), reference number [32,33,34,35]. The population data presented in this study are openly available in the United Nations Population Division’s World Population Prospects (2019 Revision) at https://population.un.org/wpp/Download/Standard/CSV/ (accessed on 19 May 2022), reference number [39].

## Supplementary Materials

The following supporting information can be downloaded at: https://www.mdpi.com/article/10.3390/healthcare10091611/s1, Table S1: Incidence, age, period and cohort data from 1990 to 2019; Table S2: Incidence data matrix by age group from 1990 to 2019; Table S3: Population data matrix by age group from 1990 to 2019; Table S4: Incidence, death and DALY data from 1990 to 2019; Table S5: The contributed data of smoking, alcohol use and chewing tobacco to death and DALY respectively from 1990 to 2019; Table S6: The contributed data of smoking and alcohol use to death and DALY respectively by age group in 2019; Table S7: Predicted data on the incidence in China from 2020 to 2049.

## Appendix A

The calculation formula of APC is ${\mathrm{APC}}_{i}=\left[\left(\exp\left(\beta_{i}\right)-1\right)\right]\times 100$, where $\beta_{i}$ represents the slope of the trend segment. We also calculated the AAPC during the entire observation period, which can report the overall trends over our study period. APC > 0 means that the rate increases yearly, APC < 0 means that the rate decreases yearly; AAPC > 0 means that the rate increases on average every year with a specific AAPC value, and AAPC < 0 means that the rate decreases on average every year. If APC = AAPC, then the trend curve has no connection points, indicating that the data as a whole presents a monotonically increasing or decreasing trend.

## Appendix B

The APC model is based on a Poisson distribution, which improves the traditional descriptive analysis method. It decomposes the target analysis variables from three dimensions of age, period and cohort, allowing to better report the risk of disease onset. The basic expression is: $\ln incidence=\mu +\alpha_{a}+\beta_{b}+\gamma_{c}+\varepsilon_{abc}$, where lnincidence represents the natural logarithm of the incidence of Alzheimer’s disease in China, μ is the intercept term, $\alpha_{a}$ is the age effect in the a age group, $\beta_{b}$ is the period effect in the b age group, $\gamma_{c}$ is the cohort effect of the c birth cohort, and $\varepsilon_{abc}$ is the error term or residual term.

As age, period and cohort have a completely linear relationship, there is a problem that the model cannot be recognized. Existing studies have adopted different approaches to solve this multicollinearity problem, such as the two-factor model, penalty function method, instrumental variable method and so on. The intrinsic Estimator (IE algorithm) was used in this study. The endogenous factor method was proposed by Fu and Yang et al. It does not require the researchers to make model assumptions in advance and has the characteristics of estimability and unbiasedness.

## Appendix C

The Bayesian analysis method provides a method to calculate the probability of hypothesis—that is, according to the Bayesian formula, the prior information about unknown parameters and sample information are integrated to obtain posterior information, and then unknown parameters are inferred according to the posterior information. Among them, prior information comes from previous statistical conclusions, experiences or assumptions. Its formula is expressed as follows:

$$P\left(A_{i}\right)|B=\frac{P\left(A_{i}\right)P\left(B|A_{i}\right)}{\sum_{i}^{n}P\left(A_{i}\right)P\left(B|A_{i}\right)}\left(i=1,2,\ldots n\right)$$

where $A_{1}$, $A_{2}$, … $A_{n}$ are the complete event group of incompatible sample space, $P\left(A_{i}\right)\;$> 0, *P*(*B*) > 0.

The above formula is called the Bayes’ formula, where $P\left(A_{i}\right)$ represents the probability of the occurrence of $A_{i}$—namely, the prior probability. This probability is a known probability, and thus the conditional probability of B occurring under the condition of $A_{i}$ occurring can be calculated according to the sample information—that is, $P\left(B|A_{i}\right)$. Then, the probability $P\left(A_{i}\right)|B$ of the occurrence of condition *A* can be calculated according to the Bayesian formula under the condition of the occurrence of the resulting event *B*. This probability is the probability determined after the test—that is, the posterior probability.

It is difficult to determine the posteriori probability distribution type when applying the Bayes formula, especially when the posteriori probability distribution is complicated. With the development of computers, the method based on MCMC simulation can solve the problem that the posterior probability is difficult to determine. The MCMC method is to obtain the approximate solution of the problem by simulating random events repeatedly and then statistically analyzing the simulation results.

Therefore, the error can be reduced by increasing the simulation times. When solving APC model parameters, the method based on MCMC simulation can be considered. When solving APC model parameters by MCMC simulation, the distribution of each parameter can be set according to the situation of sample data. By smoothing the effects of age, period and cohort, large fluctuations between two adjacent groups can be avoided in the estimation results, making the estimation results more robust and reliable.

The BAMP software package can be used for modeling and prediction. The BAMP software was written by Volker Sehmid and Leonhard Held based on a Bayesian age-period method. Queue models are used to analyze and predict illness or death in software. The software can also be applied to data types with different ages and periods. The principle of software simulation is to use Markov chain Monte Carlo method to iterate, so that the posterior probability distribution converges to a relatively stable state and then to estimate the parameter values through the estimated samples obtained by iteration. Therefore, the more iterations, the higher the accuracy of model fitting. Generally, the number of iterations set in the study is 1.01 million, and the first 10,000 results of the initial iteration are omitted to eliminate the influence of artificially set initial values on the results. After that, one result is selected for every 500 iterations to form the sample of parameter estimation.

## References

[1] Siakholak F.R., Ghoncheh M., Pakzad R., Gandomani H.S., Ghorat F., Salehiniya H. (2016). Epidemiology, incidence and mortality of oral cavity and lips cancer and their relationship with the human development index in the world. Biomed. Res. Ther..

[2] Mathavan S., Kue C.S., Kumar S. (2021). Identification of potential candidate genes for lip and oral cavity cancer using network analysis. Genom. Inform..

[3] Mathavan S., Kumar S. Identification of potential biomarkers for lip and oral cavity cancer using systems biology approach. Proceedings of the Asian Regional Conference on Systems Biology (ARCSB2020).

[4] Pereira M.C., Oliveira D.T., Landman G., Kowalski L.P. (2007). Histologic subtypes of oral squamous cell carcinoma: Prognostic relevance. J. Can. Dent. Assoc..

[5] Stnga A.C., Mrgritescu O., Stng A.S., Pirici D., Cruce M. (2011). VEGFR1 and VEGFR2 immunohistochemical expression in oral squamous cell carcinoma: A morphometric study. Rom. J. Morphol. Embryol..

[6] Miranda-Filho A., Bray F. (2020). Global patterns and trends in cancers of the lip, tongue and mouth. Oral Oncol..

[7] Shrestha A.D., Vedsted P., Kallestrup P., Neupane D. (2019). Prevalence and incidence of oral cancer in low- and middle-income countries: A scoping review. Eur. J. Cancer Care.

[8] Ferlay J., Colombet M., Soerjomataram I., Dyba T., Randi G., Bettio M., Gavin A., Visser O., Bray F. (2018). Cancer incidence and mortality patterns in Europe: Estimates for 40 countries and 25 major cancers in 2018. Eur. J. Cancer.

[9] Warnakulasuriya S. (2009). Global epidemiology of oral and oropharyngeal cancer. Oral Oncol..

[10] Priya M., Lando H.A. (2015). Tobacco control: An issue twinned with oral cancer control. Int. Dent. J..

[11] Salehiniya H., Raei M. (2020). Oral cavity and lip cancer in the world: An epidemiological review. Biomed. Res. Ther..

[12] Gupta B., Bray F., Kumar N., Johnson N.W. (2017). Associations between oral hygiene habits, diet, tobacco and alcohol and risk of oral cancer: A case–control study from India. Cancer Epidemiol..

[13] Moore S.R., Johnson N.W., Pierce A.M., Wilson D.F. (2010). The epidemiology of lip cancer: A review of global incidence and aetiology. Oral Dis..

[14] Radoi L., Luce D. (2012). A review of risk factors for oral cavity cancer: The importance of a standardized case definition. Community Dent. Oral Epidemiol..

[15] Merchant A., Husain S., Hosain M., Fikree F.F., Saeed S.A. (2000). Paan without tobacco: An independent risk factor for oral cancer. Int. J. Cancer.

[16] Ribeiro I.L.A., de Medeiros J.J., Rodrigues L.V., Valença A.M.G., Neto E.D.A.L. (2015). Factors associated with lip and oral cavity cancer. Rev. Bras. De Epidemiol..

[17] Freedman N.D., Park Y., Subar A.F., Albert R. (2008). Fruit and vegetable intake and head and neck cancer risk in a large United States prospective cohort study. Int. J. Cancer.

[18] Bravi F., Bosetti C., Filomeno M., Levi F., Garavello W., Galimberti S., Negri E., LaVecchia C. (2013). Foods, nutrients and the risk of oral and pharyngeal cancer. Br. J. Cancer.

[19] Franceschi S., Favero A., Conti E., Talamini R., Volpe R., Negri E., Barzan L., Vecchia C.L. (1999). Food groups, oils and butter, and cancer of the oral cavity and pharynx. Br. J. Cancer.

[20] Hashibe M., Sankaranarayanan R., Thomas G., Kuruvilla B., Mathew B., Somanathan T., Parkin D.M., Zhang Z.F. (2000). Alcohol drinking, body mass index and the risk of oral leukoplakia in an Indian population. Int. J. Cancer.

[21] Visscher J., Waal I. (1998). Etiology of cancer of the lip. Int. J. Oral Maxillofac. Surg..

[22] Brown L.M., Gridley G., Diehl S.R., Winn D.M., Harty L.C., Otero E.B., Fraumeni J.F., Hayes R.B. (2001). Family cancer history and susceptibility to oral carcinoma in Puerto Rico. Cancer.

[23] Bräutigam K., Meier S., Meneder S., Proppe L., Stroschein K., Polack S., Köster F., Rody A., Baum S. (2022). Distribution of HPV Subtypes in Diverse Anogenital and Oral Samples from Women and Correlation of Infections with Neoplasia of the Cervix. Cancers.

[24] Rosenblatt K.A. (2004). Marijuana Use and Risk of Oral Squamous Cell Carcinoma. Cancer Res..

[25] Kreimer A.R., Viscidi R., Pawlita M., Fakhry C., Koch W.M., Westra W.H., Gillison M.L. (2007). Case-control study of human papillomavirus and oropharyngeal cancer. Mass. Med. Soc..

[26] Giovenco D.P., Spillane T.E., Maggi R.M., Lee E.Y., Philbin M.M. (2021). Multi-level drivers of tobacco use and purchasing behaviors during COVID-19 “lockdown”: A qualitative study in the United States. Int. J. Drug Policy.

[27] Grossman E.R., Benjamin-Neelon S.E., Sonnenschein S. (2020). Alcohol Consumption during the COVID-19 Pandemic: A Cross-Sectional Survey of US Adults. Int. J. Environ. Res. Public Health.

[28] Robinson E., Boyland E., Chisholm A., Harrold J., Maloney N.G., Marty L., Mead B.R., Noonan R., Hardman C.A. (2020). Obesity, eating behavior and physical activity during COVID-19 lockdown: A study of UK adults—ScienceDirect. Appetite.

[29] Ruiz-Roso M.B., Padilha P., Mantilla-Escalante D.C., Ulloa N., Dávalos A. (2020). Covid-19 Confinement and Changes of Adolescent’s Dietary Trends in Italy, Spain, Chile, Colombia and Brazil. Nutrients.

[30] Nath B.S., Ferreira B.J., McVicar B.A., Oshilaja B.T., Dds B.S. (2022). Rise in oral cancer risk factors associated with the COVID-19 pandemic mandates a more diligent approach to oral cancer screening and treatment. J. Am. Dent. Assoc..

[31] Tiyuri A., Mohammadian-Hafshejani A., Iziy E., Gandomani H.S., Salehiniya H. (2017). The incidence and mortality of lip and oral cavity cancer and its relationship to the 2012 Human Development Index of Asia. Biomed. Res. Ther..

[32] Li N., Deng Y., Zhou L., Tian T., Yang S., Wu Y., Zheng Y., Zhai Z., Hao Q., Song D. (2019). Global burden of breast cancer and attributable risk factors in 195 countries and territories, from 1990 to 2017: Results from the Global Burden of Disease Study 2017. J. Hematol. Oncol..

[33] Zhai Z., Zheng Y., Li N., Deng Y., Wang Z. (2020). Incidence and disease burden of prostate cancer from 1990 to 2017: Results from the Global Burden of Disease Study 2017: Global disease burden of prostate cancer. Cancer.

[34] Zhou L., Deng Y., Li N., Zheng Y., Dai Z. (2019). Global, regional, and national burden of Hodgkin lymphoma from 1990 to 2017: Estimates from the 2017 Global Burden of Disease study. J. Hematol. Oncol..

[35] Deng Y., Wang M., Zhou L., Zheng Y., Dai Z. (2020). Global burden of larynx cancer, 1990-2017: Estimates from the global burden of disease 2017 study. Aging.

[36] Qu Y., Wang T., Yang J., Zhang J., Lyu J. (2019). GBD database application and data extraction methods and processes. Chin. J. Evid.-Based Cardiovasc. Med..

[37] Dereje N. (2020). Global burden of 369 diseases and injuries in 204 countries and territories, 1990–2019: A systematic analysis for the Global Burden of Disease Study 2019. Lancet.

[38] GBD 2019 Risk Factors Collaborators (2021). Global burden of 87 risk factors in 204 countries and territories, 1990–2019: A systematic analysis for the Global Burden of Disease Study 2019. Lancet.

[39] United Nations World Population Prospects 2019 Highlights. https://population.un.org/wpp/Download/Standard/CSV/.

[40] Kim H.J., Fay M.P., Feuer E.J., Midthune D.N. (2000). Permutation tests for joinpoint regression with applications to cancer rates. Stat. Med..

[41] Zhu W., Wang Y., Li T., Chen W., Wang W. (2021). Gap to End-TB targets in eastern China: A joinpoint analysis from population-based notification data in Zhejiang Province, China, 2005–2018. Int. J. Infect. Dis..

[42] Bramajo O.N., Valk H. (2022). An Age-Period-Cohort Approach to Analyse Late-Life Depression Prevalence in Six European Countries, 2004–2016. Eur. J. Popul..

[43] Luo G., Zhang Y., Guo P., Wang L., Huang Y., Li K. (2017). Global patterns and trends in stomach cancer incidence: Age, period and birth cohort analysis. Int. J. Cancer.

[44] Wilkinson K., Righolt C.H., Elliott L.J., Fanella S., Mahmud S.M. (2022). The impact of pertussis vaccine programme changes on pertussis disease burden in Manitoba, 1992–2017—An age-period-cohort analysis. Int. J. Epidemiol..

[45] Riebler A., Held L. (2017). Projecting the future burden of cancer: Bayesian age–period–cohort analysis with integrated nested Laplace approximations. Biom. J..

[46] He X., Zhang W. (2021). China’s Changing Population Structure and Its Implications for US Agricultural Exports.

[47] Hussein A.A., Helder M.N., de Visscher J.G., Leemans C.R., Braakhuis B.J., de Vet H.C., Forouzanfar T. (2017). Global incidence of oral and oropharynx cancer in patients younger than 45 years versus older patients: A systematic review. Eur. J. Cancer.

[48] Kishore C., Prashanti B. (2021). 1375 Age-standardization to world population and under-estimation of oral cancer burden. Int. J. Epidemiol..

[49] Batchelor P. (2015). The changing epidemiology of oral diseases in the elderly, their growing importance for care and how they can be managed. Age Ageing.

[50] Gómez I., Seoane J., Varela-Centelles P., Diz P., Takkouche B. (2010). Is diagnostic delay related to advanced-stage oral cancer? A meta-analysis. Eur. J. Oral Sci..

[51] Varela-Centelles P. (2022). Early Diagnosis and Diagnostic Delay in Oral Cancer. Cancers.

[52] Feng X.F., Huang H.T., Wang R. (2016). Study on the Patient Delay among Oral Cancer Patients. J. Oral Sci. Res..

[53] Luo Y., Su B., Zheng X. (2021). Trends and Challenges for Population and Health During Population Aging—China, 2015–2050. China CDC Wkly..

[54] Yang Y., Land K.C. (2006). A mixed models approach to the age-period-cohort analysis of repeated cross-section surveys, with an application to data on trends in verbal test scores. Sociol. Methodol..

[55] Geng Y., Zhao L., Wang Y., Jiang Y., Meng K., Zheng D., Lin C.P. (2018). Competency model for dentists in China: Results of a Delphi study. PLoS ONE.

[56] Dhillon P.K., Mathur P., Nandakumar A., Fitzmaurice C., Kumar G.A., Mehrotra R., Shukla D.K., Rath G.K., Gupta P.C., Swaminathan R. (2018). The burden of cancers and their variations across the states of India: The Global Burden of Disease Study 1990–2016. Lancet Oncol..

[57] Shield K.D., Ferlay J., Jemal A., Sankaranarayanan R., Chaturvedi A.K., Bray F., Soerjomataram I. (2017). The global incidence of lip, oral cavity, and pharyngeal cancers by subsite in 2012. CA Cancer J. Clin..

[58] Keyuri P., McQueen K., Feeley T.W. (2015). The Global Burden of Cancer 2013. JAMA Oncol..

[59] Ren Z., Hu C., Lyu J., Ji T. (2019). Global, Regional, and National Burdens of Oral Cancer, 1990 to 2017: Results from the Global Burden of Disease Study. Cancer Commun..

[60] Gu J., Song J.W., Liu Y., Hui R., Liu Y. (2022). Diease burden and trend of oral cancer in China from 1990–2019. Chin. Prev. Med..

[61] Ren Z.H., Hu C.Y., He H., Li Y., Lyu J. (2020). Global and regional burden of oral cancer from 1990 to 2017: A report on the Global Burden of Disease Study. Chin. J. Cancer Res..

[62] Song S.W., Xie L., Huang P. (2022). Analysis and prediction of mortality risk of lip and oral cavity cancer in China, 1990–2019. Mod. Prev. Med..

[63] Park J.-O., Nam I.-C., Kim C.-S., Park S.-J., Lee D.-H., Kim H.-B., Han K.-D., Joo Y.-H. (2022). Sex Differences in the Prevalence of Head and Neck Cancers: A 10-Year Follow-Up Study of 10 Million Healthy People. Cancers.

[64] Mehrtash H., Duncan K., Parascandola M., David A., Gritz E.R., Gupta P.C., Mehrotra R., Nordin A.S.A., Pearlman P.C., Warnakulasuriya S. (2017). Defining a global research and policy agenda for betel quid and areca nut. Lancet Oncol..

[65] Warnakulasuriya S. (2009). Causes of oral cancer—An appraisal of controversies. Br. Dent. J..

[66] Winn D.M., Lee Y.A., Hashibe M., Boffetta P., The INHANCE Consortium (2015). The INHANCE consortium: Toward a better understanding of the causes and mechanisms of head and neck cancer. Oral Dis..

[67] Sung W.W., Hsu Y.C., Dong C., Chen Y.C., Chen C.J. (2021). Favorable Lip and Oral Cancer Mortality-to-Incidence Ratios in Countries with High Human Development Index and Expenditures on Health. Int. J. Environ. Res. Public Health.

[68] Gupta B., Johnson N., Kumar N. (2016). Global Epidemiology of Head and Neck Cancers: A Continuing Challenge. Oncology.

[69] Yang M.J., Chung C.Y., Kuo C.C., Ho C.K. (1996). and Kawachi, I. The influence of work nature and workplace subculture on individual drinking behavior: An exploratory pilot study. Kaohsiung J. Med. Sci..

[70] Clegg D., Hevener A.L., Moreau K.L., Morselli E., Criollo A., Van Pelt R.E., Vieira-Potter V.J. (2017). Sex Hormones and Cardiometabolic Health: Role of Estrogen and Estrogen Receptors. Endocrinology.

[71] Kujan O., Oliver R., Thakker N., Sloan P., Glenny A.-M. (2009). Screening programmes for the early detection and prevention of oral cancer. Aust. Dent. J..

[72] Warnakulasuriya S., Kerr A.R. (2021). Oral Cancer Screening: Past, Present, and Future. J. Dent. Res..

[73] Peng Y., Liu J., Xu K., Liu X., Wang J., Liao X. (2019). Incidence and Mortality of Oral Cancer in Registered Regions of Hunan in 2009–2015. China Cancer.

[74] Liu G., Yang L., Fu Z., Xu A., Guo X. (2019). Incidence and mortality of oral cancer in Shandong Province in 2014. J. Shandong Univ..

[75] Bai X., Bai X., Zhao D., Zhang J. (2017). Analysis of primary oral squamous cell carcinoma in Beijing inhabitants: A 5-year continuous study of a single center. J. Pract. Stomatol..

